# Telehealth for Sexual and Reproductive Healthcare: Evidence Map of Effectiveness, Patient and Provider Experiences and Preferences, and Patient Engagement Strategies

**DOI:** 10.3390/clinpract16010014

**Published:** 2026-01-09

**Authors:** Romil R. Parikh, Nishka U. Shetty, Chinar Singhal, Prachi Patel, Priyanka Manghani, Ashwin A. Pillai, Luz Angela Chocontá-Piraquive, Mary E. Butler

**Affiliations:** 1 Division of Health Policy & Management, University of Minnesota School of Public Health, Minneapolis, MN 55455, USA; 2 Kymera Medical Group, Eastern New Mexico Medical Center, Roswell, NM 88201, USA; 3 University of Minnesota Hubert H. Humphrey School of Public Affairs, Minneapolis, MN 55455, USA; 4 Department of Family Medicine and Community Health, University of Minnesota Medical School, Minneapolis, MN 55455, USA; 5 Bergen New Bridge Medical Center, Paramus, NJ 07652, USA; 6 Division of HIV, Infectious Diseases & Global Medicine, University of California, San Francisco, CA 94143, USA; 7 Department of Internal Medicine, University of Connecticut School of Medicine, Farmington, CT 06032, USA

**Keywords:** telehealth, sexual and reproductive health, HIV, family planning, clinical practice, telemedicine

## Abstract

Objective: The aim of this study was to systematically map evidence to inform best practices for sexual and reproductive healthcare delivered via telehealth (TeleSRH) in United States-based Title X-funded clinics. Methods: We searched three databases (2017–2025) for studies evaluating effectiveness, harms, patient and provider experiences, barriers/facilitators, and engagement strategies encompassing TeleSRH for sexually transmitted infections (STIs), contraceptive care/family planning (CC/FP), and sexual wellness, in countries with a human development index of ≥0.8. Results: From 5963 references and 436 articles, we included 142 eligible publications. TeleSRH use declined since the COVID-19 pandemic’s peak but remains higher than pre-pandemic. Evidence comes mostly from poor-quality studies. TeleSRH increases access and adherence to STI prevention (e.g., pre-exposure prophylaxis for HIV). Tele-follow-up may safely facilitate HIV care continuity. For CC/FP, TeleSRH is comparable to in-person care for patient satisfaction and uptake; patients are less likely to select long-acting reversible contraception but post-initiation tele-follow-up may increase its continuation rates. Vasectomy completion rates may be similar between pre-procedural counseling via telehealth versus in-person. TeleSRH’s potential benefits might include reduced travel time, wait times, no-show rates, and clinic human resource burden (via tele-triaging) and increased preventative screening rates for STIs and non-communicable diseases, prescription refill rates, ability to receive confidential care in preferred settings, and rural/marginalized community outreach. Implementation challenges span technological and capital constraints, provider availability, staff capability building, restrictive policies, language incompatibility, and patient mistrust. Supplementing synchronous TeleSRH with asynchronous communication (e.g., mobile application) may improve continued patient engagement. Conclusions: Preventive, diagnostic, and therapeutic TeleSRH can be effective, with high patient acceptability; however, effectiveness and adoption hinge on contextual factors outlined in this review.

## 1. Introduction

Sexual and reproductive healthcare (SRH) is a cornerstone of preventive health, encompassing a wide range of services including sexually transmitted infection (STI) prevention and treatment, contraceptive counseling, family planning, and the promotion of sexual well-being [1]. Access to timely, high-quality SRH services is essential for individual health and public health outcomes alike [1,2]. However, long-standing barriers such as geographic isolation, provider shortages, transportation challenges, stigma, and cost continue to limit access, particularly among underserved populations in the United States [1,2,3].

Telehealth has emerged as a promising strategy to expand access to SRH services, particularly within Title X-funded settings that serve individuals regardless of ability to pay [4,5]. With the COVID-19 pandemic catalyzing widespread adoption of virtual care models, SRH services rapidly adapted to incorporate telehealth via synchronous modalities such as video and telephone and asynchronous modalities such as patient portals and mobile applications [4,6]. Telehealth offers potential advantages, including increased privacy, convenience, and reach, especially for populations facing structural barriers to in-person care [7,8,9,10,11,12,13,14]. Despite its promise, questions persist about its effectiveness, acceptability, and equity [4]. Furthermore, the existing literature varies in scope, populations studied, and the definitions of SRH services employed, making it challenging for clinicians, healthcare administrators, and policymakers to synthesize best practices and identify implementation gaps [4,5,6,7]. To date, there is limited comprehensive mapping of the landscape of telehealth applications for SRH that focuses on a wider range of services covered by Title X-funded clinics, such as services for STI, contraceptive care, family planning, and sexual well-being.

Previously, a systematic review evaluated telehealth services for women’s preventive health services, which included STI prevention and family planning services, in studies published between July 2016 and March 2022 [4]. This review found only two randomized controlled trials and five non-randomized studies which met their eligibility criteria. However, Title X-funded clinics cover a wider range of services (e.g., post-diagnostic management and treatment of STI, sexual well-being, etc.) and cater to a wider consumer base apart from only biologically female patients. Implementation of telehealth or any modifications to clinic workflows would require considering its impact on all patient populations served by the clinic. Therefore, there is need for a comprehensive evidence review spanning all services provided in such clinics and for all patient populations served in such clinics.

This review aims to identify and describe the current research on sexual and reproductive health services provided via telehealth in developed countries, for the intended target audience being Title-X funded clinics. For this evidence map, we operationalized “sexual and reproductive healthcare” as the subset of services that align most closely with the U.S. Title X program and with SRH domains most commonly delivered through telehealth in clinical practice. These include family planning/contraception counseling and provision, STI prevention, testing, and management, including HIV prevention and care, and sexual wellness services (e.g., management of sexual dysfunction, libido concerns, or partner communication). Broader gynecologic conditions such as menstrual disorders, dysmenorrhea, pelvic pain, and other non-SRH gynecologic diagnoses were excluded because their evaluation and management typically rely on in-person diagnostic procedures and fall outside the primary scope of Title X-funded care. Our search strategy was therefore tailored to focus on telehealth-enabled SRH services most relevant to U.S. public health and clinical delivery contexts.

We restrict this mapping review to countries with a human development index of 0.8 or more. This threshold corresponds to very-high-HDI settings, which more closely resemble U.S. Title X clinical environments in terms of digital infrastructure, reimbursement policies, workforce capacity, and patient population characteristics. Including lower-HDI settings would have introduced heterogeneity in telehealth feasibility, regulation, and access that may not be generalizable to U.S. Title X clinics. While studies from high-HDI countries (HDI 0.7–0.79; example, Mexico, Peru, Colombia, Ecuador, etc.) may also offer relevant insights, they were excluded to maintain fidelity to the review’s implementation-focused purpose for U.S. Title X-funded settings. We highlight this as an area for expansion in future evidence reviews. This review may be used as a resource for implementation of telehealth in healthcare settings and for the development of practice protocols, policy recommendations, and other related work. The second purpose of this review is to identify research gaps and inform future research on sexual and reproductive health delivered via telehealth.

### Key Questions

Five key questions were identified to guide this mapping review. The literature reviewed in this report is organized around these questions. The five key questions are as follows:

1. How have effectiveness, harms, and acceptability of telehealth methods for SRH been studied?
2. What are patient experiences, patient preferences, and patient choice in the context of telehealth utilization?
3. What are provider experiences and preferences in the context of telehealth utilization?
4. What are the barriers to and facilitators of telehealth methods for sexual and reproductive healthcare services?
5. What are the patient engagement strategies for telehealth?

## 2. Methods

We conducted our mapping review and report our methods and findings based on the methodology for evidence maps and the Preferred Reporting Items for Systematic Review and Meta-Analyses (PRISMA) guidance for scoping reviews [15,16]. We registered our protocol on the Open Science Framework registry (https://doi.org/10.17605/OSF.IO/ZTX7R).

### 2.1. Selection Criteria

We developed our selection criteria based on the PICOTS framework; these criteria are shown in Table 1 [16]. Briefly, we included primary research studies for teleSRH in countries with HDI 0.8 or more published between 2017 and 2025. We used two broad conceptual definitions for SRH and telehealth, as described in Appendix A. In this review, we restricted our scope to telehealth involving only bi-directional communication between a provider and a patient. We included studies reporting at least one outcome related to SRH.

### 2.2. Peer-Reviewed Literature Search and Screening

We developed a search strategy with the help of a research librarian, following the Peer Review of Electronic Search Strategies (PRESS) guideline [17]. The search combined the two broad concepts as such: (sexual and reproductive healthcare services) AND (telehealth). For the concept of sexual/reproductive healthcare services, these specific concepts were used: (contraception OR family planning OR gynecology OR women’s health OR sexual health OR sexual wellness OR reproductive health OR sexually transmitted diseases OR fertility OR abortion OR unplanned pregnancy OR unwanted pregnancy OR preconception OR pre-pregnancy OR Papillomavirus vaccines). For the concept of telehealth, these specific concepts were used: (telemedicine OR telehealth OR telephone OR smartphone OR cellular device OR mobile device OR text messaging OR SMS OR virtual OR remote monitoring OR ehealth OR mhealth OR mobile health OR digital health). Telehealth was required to be a key concept by using title keywords or focused subject headings, and sexual/reproductive health was accepted as a general concept by using any subject heading or multi-placement keywords (title, abstract, etc.). Results were limited to journal articles in the English language, published between January 2017 and January 2025, to focus on recent evidence that may be applicable to current systems and as a continuation of a previous evidence map which included studies published through 2016 and the AHRQ systematic review that used a similar cut-off citing the same reason [4,5]. Searches were executed in three electronic databases: Medline (Ovid), Embase (Ovid), and CINAHL (Ebsco). The query used both keywords and subject headings for each concept, and a separate strategy was developed and optimized for each electronic database. The complete search strategies are available in Appendix B. All searches were conducted on 25 January 2025.

We uploaded citations to the PICO Portal™ (www.picoportal.net), an online tool for systematic reviews, and we removed duplicates using its software [18]. PICO Portal uses machine learning to prioritize citations most likely to meet eligibility criteria. Initially, two independent reviewers assessed titles and abstracts for relevance to the key questions based on predefined inclusion and exclusion criteria (Table 1). Any disagreements were resolved through team discussions. Once the algorithm achieved a 99% recall rate for eligible citations, we switched to one reviewer until a 100% recall rate and zero false negatives were achieved. Two independent reviewers then screened each article at the full-text level. Any disagreements were resolved by group discussion.

### 2.3. Data Management, Risk of Bias Assessment, and Synthesis

Data from included studies were extracted using Microsoft Excel [Microsoft Corporation. (2024)] sheets by one reviewer and checked for accuracy by a second reviewer (Supplemental Tables S1 and S2). We extracted the following data elements: study id, title, lead author contact details, geographical setting, study aim(s), study design, study period, participant characteristics (e.g., total *N*, population descriptions, age, race, ethnicity, etc.), service or intervention details, and summary of results. We focused only on outcomes directly related to sexual and reproductive health services, for example, rates for STI screening, STI treatment, STI incidence, STI prescription refill rates, rate of contraception uptake, relevant lab results (e.g., viral load), compliance and adherence to prescriptions, and process outcomes such as travel time, wait times, no-show rates, ability to receive confidential care in preferred settings, and rural/marginalized community outreach, etc.

In this review, we assessed the risk of bias (RoB) in the included RCTs using the Cochrane RoB-2 tool and in included cohort and quasi-experimental studies using the ROBINS-I tool [16,18]. For the ROBINS-I tool, full assessments were to be conducted only for studies which passed the pre-screener portion of the tool and were not assessed with critical RoB for the selection bias domain.

Data were analyzed descriptively in Microsoft Excel [Microsoft Corporation (2024), Redmond, WA, USA] and summarized narratively because the uniqueness of each telehealth intervention hindered quantitative pooling. Tableau [Salesforce (2025), Seattle, WA, USA] was used for data visualization. Narrative summaries presented in the Results Section are organized around the five key questions guiding the report.

## 3. Results

**Figure 1:**
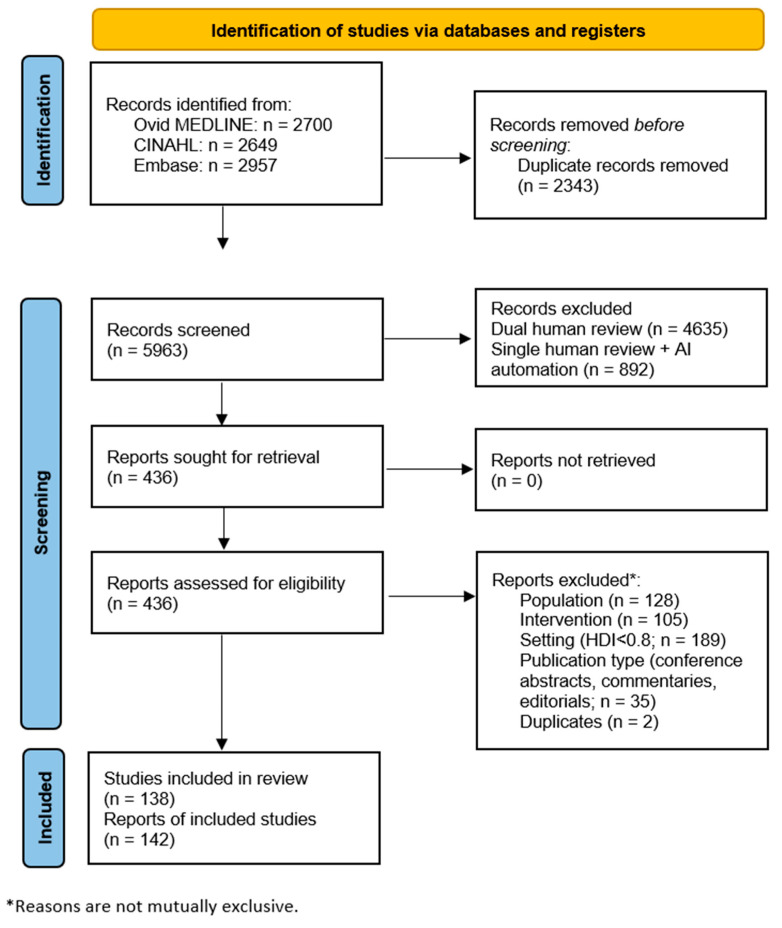
PRISMA Flow Diagram for Study Selection. AI—artificial intelligence-assisted; HDI—human development index.

Searches resulted in 8306 references (Ovid MEDLINE: 2700 references; CINAHL: 2649 references; and Embase: 2957 references). The PRISMA flow diagram for screening and selection is presented in Figure 1. After deduplication, the initial sample totaled 5963 references for dual screening of title and abstract. In the full-text screening phase, 436 references were screened. A total of 142 references met study inclusion/exclusion criteria after the full-text screening [19,20,21,22,23,24,25,26,27,28,29,30,31,32,33,34,35,36,37,38,39,40,41,42,43,44,45,46,47,48,49,50,51,52,53,54,55,56,57,58,59,60,61,62,63,64,65,66,67,68,69,70,71,72,73,74,75,76,77,78,79,80,81,82,83,84,85,86,87,88,89,90,91,92,93,94,95,96,97,98,99,100,101,102,103,104,105,106,107,108,109,110,111,112,113,114,115,116,117,118,119,120,121,122,123,124,125,126,127,128,129,130,131,132,133,134,135,136,137,138,139,140,141,142,143,144,145,146,147,148,149,150,151,152,153,154,155,156,157,158,159,160].

### 3.1. Results of the Literature Search

**Figure 2:**
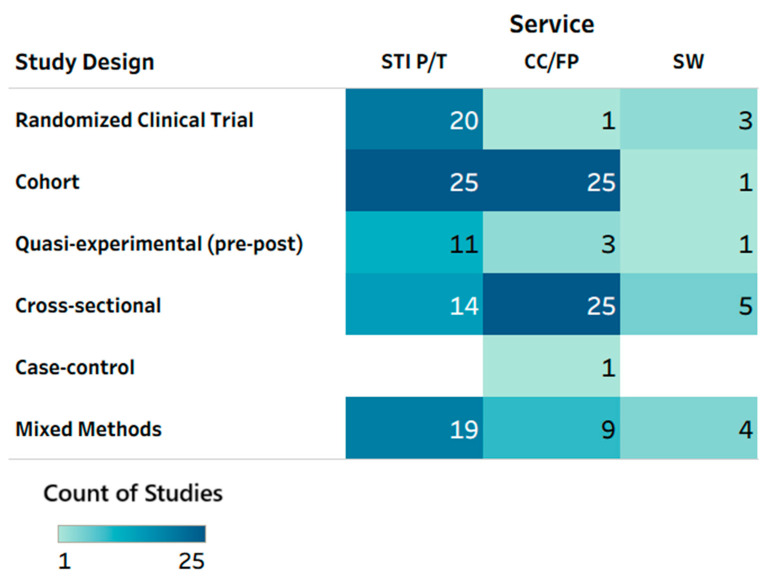
Heat map: service type by study design. CC/FP—contraceptive care or family planning; STI P/T—sexually transmitted infection prevention and treatment; SW—sexual well-being. Note: Multiple service types could have been tested in a given study/publication, so total counts may not add up to the total number of reports included in this scoping review (*n* = 142 publications).

A total of 138 unique studies in 142 publications [19,20,21,22,23,24,25,26,27,28,29,30,31,32,33,34,35,36,37,38,39,40,41,42,43,44,45,46,47,48,49,50,51,52,53,54,55,56,57,58,59,60,61,62,63,64,65,66,67,68,69,70,71,72,73,74,75,76,77,78,79,80,81,82,83,84,85,86,87,88,89,90,91,92,93,94,95,96,97,98,99,100,101,102,103,104,105,106,107,108,109,110,111,112,113,114,115,116,117,118,119,120,121,122,123,124,125,126,127,128,129,130,131,132,133,134,135,136,137,138,139,140,141,142,143,144,145,146,147,148,149,150,151,152,153,154,155,156,157,158,159,160] were included in the review [Supplemental Table S1]. The most common study designs were cohort studies (k = 48), cross-sectional studies (k = 34), mixed-methods studies (k = 23), and RCTs (k = 23). There were thirteen pre–post studies and one case–control study. Overall, as shown in Figure 2, contraceptive care and family planning was addressed in 64 studies; sexually transmitted infection prevention and treatment in 89 studies; and sexual wellness in 14 studies. Most of the RCTs (20 out of 23) focused on STI prevention or treatment [Figure 2]. Sexual wellness was the least evaluated service type, with only three RCTs and 9.9% of total studies (k = 14; Figure 2). Contraceptive care and family planning services were mostly evaluated in observational cohort (k = 25) and cross-sectional survey (k = 25) studies, with only one RCT evaluating this service type.

Most studies were conducted in the United States (106), with the majority of non-US contributions from the United Kingdom, Australia, and Canada. Among U.S.-based studies, all regional locations (the Northeast, South, Midwest, and West) had similar representation, with several studies covering multiple or unspecified regions. Patient rurality was described as urban in 31 studies, rural in 4 studies, and some combination of rural and urban and/or suburban in 16 studies; however, 83 studies did not report rurality status. Sexual and gender minority (LGBTQ+) representation was explicitly included in 41.5% studies [k = 59; Supplemental Table S1]. Among the 142 included publications, the majority reported the distribution of racial or ethnic groups in their study sample. At least 112 studies reported including racially/ethnically diverse populations, such as Black, White, Hispanic, Asian, Indigenous, Native American, Pacific Islander, “some other race”, or multiracial subgroups. These findings indicate that while studies varied widely in geography, design, and sample composition, many incorporated key dimensions of participant diversity relevant to equity in sexual and reproductive healthcare research.

**Figure 3:**
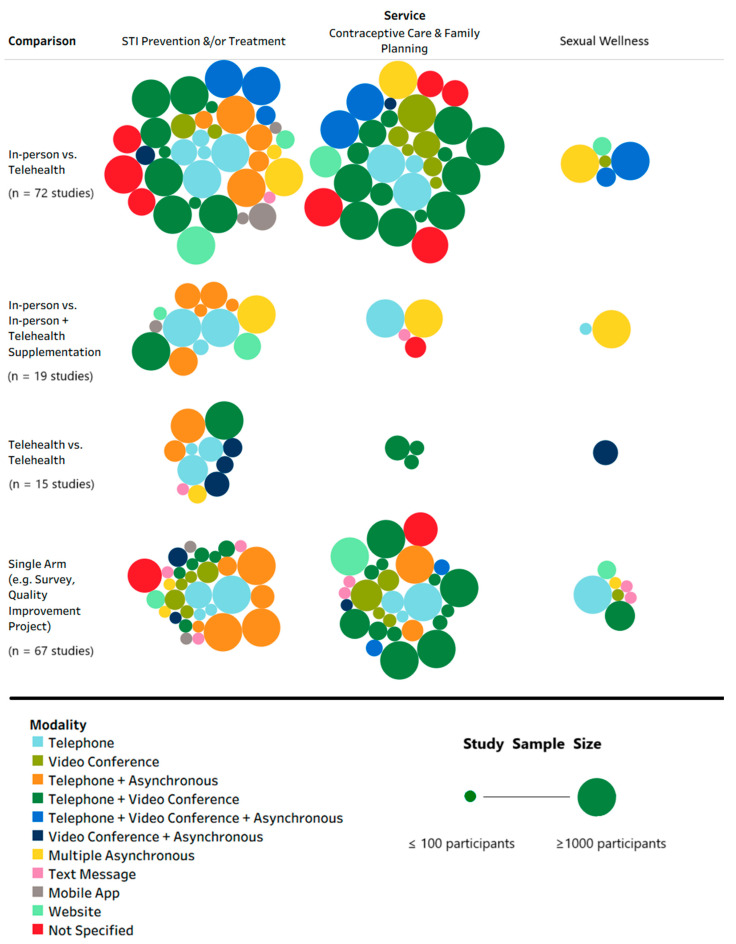
Bubble plot: service type by comparisons by mode of telehealthcare delivery. STI—sexually transmitted infection; vs.—versus. Note: Asynchronous mode includes (two-way communication between provider and patient via) text message, mobile application, website, web-based portal, and e-mail. Each bubble represents a study and the size of the bubble corresponds to study sample size which, as shown in the legend, was winsorized at a minimum of 100 participants and a maximum of 1000 participants, for visual aid. Multiple service types could have been tested in a study, and multiple types of comparisons could have been made in the same study; therefore, row totals and column totals are not presented as their sum/total would not yield the total number of reports included in this scoping review (*n* = 142 publications).

Several different modes of telehealth were evaluated across service type, individually or as combinations, including synchronous modes of communication such as telephone and videoconferencing and asynchronous modes such as text messaging, mobile application, website, web portal, and electronic mail [Figure 3].

### 3.2. Risk of Bias Assessment

**Figure 4:**
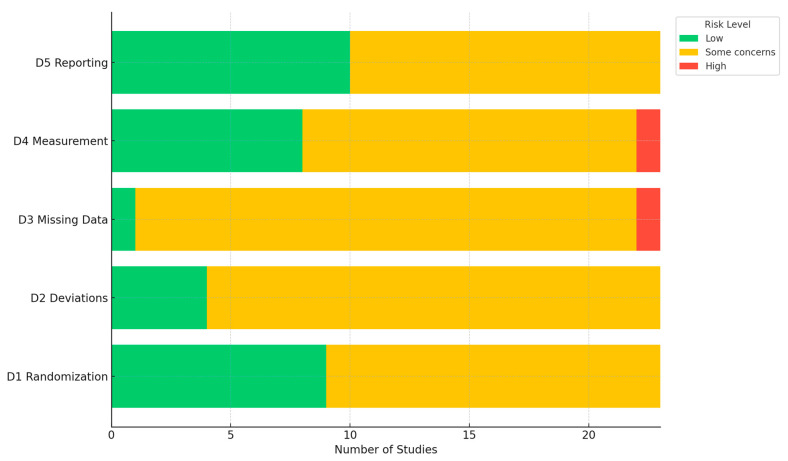
Bar graph for domain-specific risk of bias assessments. Abbreviation: D—domain; D1—bias from the randomization process; D2—bias from deviations from intended interventions; D3—bias from missing outcome data; D4—bias in the measurement of the outcome; D5—bias in the selection of the reported result.

Among RCTs, RoB ranged between moderate (some concerns) and high (Supplemental Table S3; Figure 4); common concerns, given the inherent difficulty of masking telehealth delivery, included lack of allocation concealment and challenges with blinding of participants and outcome assessors. Several trials relied on self-reported outcomes such as contraceptive adherence, satisfaction, or sexual health behaviors, which introduced potential reporting and social desirability biases. Attrition rates were often high, particularly in trials with longer follow-up periods or those targeting adolescent or marginalized populations, and few studies employed appropriate methods to address missing data. Additionally, fidelity of intervention delivery and variation in technological literacy were inconsistently assessed, limiting confidence in treatment effect estimates.

All observational cohort and quasi-experimental studies demonstrated very high susceptibility to confounding and/or selection bias, and as a result, they did not merit complete assessments using the ROBINS-I tool. Many studies used convenience samples or recruited participants already engaged with telehealth services, potentially overestimating acceptability and effectiveness. Adjustment for key covariates such as age, socioeconomic status, prior healthcare access, and digital literacy was inconsistent, and most of the studies did not employ advanced methods (e.g., propensity score matching or inverse probability weighting) to mitigate these biases. Misclassification bias was also possible where telehealth exposure was not clearly defined or where hybrid in-person/virtual models were combined. Despite these limitations, most studies provided sufficient detail on intervention characteristics and outcomes to allow synthesis; however, the overall certainty of evidence remains limited due to methodological heterogeneity and risks of bias inherent in evaluating telehealth interventions in real-world sexual and reproductive health settings.

The results of the literature search [19,20,21,22,23,24,25,26,27,28,29,30,31,32,33,34,35,36,37,38,39,40,41,42,43,44,45,46,47,48,49,50,51,52,53,54,55,56,57,58,59,60,61,62,63,64,65,66,67,68,69,70,71,72,73,74,75,76,77,78,79,80,81,82,83,84,85,86,87,88,89,90,91,92,93,94,95,96,97,98,99,100,101,102,103,104,105,106,107,108,109,110,111,112,113,114,115,116,117,118,119,120,121,122,123,124,125,126,127,128,129,130,131,132,133,134,135,136,137,138,139,140,141,142,143,144,145,146,147,148,149,150,151,152,153,154,155,156,157,158,159,160] are organized around the five key questions driving this study (i.e., *effectiveness and acceptability of telehealth methods*; *patient experiences; provider experiences*; *barriers and facilitators*; and *effectiveness of patient engagement strategies*), and study-level details are presented in Supplemental Tables S1 and S2. Within each subsection, evidence is reviewed, summarized, and synthesized as related to contraceptive care and family planning; sexually transmitted infection prevention and treatment; or sexual wellness (respectively) in alignment with the sexual and reproductive health conceptual definition guiding this report. Due to the overlap in articles discussing contraceptive care and family planning, these concepts were combined into a single construct for reporting results.

### 3.3. Key Question 1: Effectiveness, Harms, and Acceptability of Telehealth Interventions

*Contraceptive care and family planning.* Thirty-nine studies under KQ1 (KQ, key question) evaluated CC/FP delivered via telehealth. Overall, several studies examined whether teleSRH interventions improved the ability to provide access to care for people who may be missed in regular care settings. For example, one study focused on people with depression compared in-person visits and a 4-week ecological momentary intervention delivered via bi-directional text messaging to reduce sexual and reproductive health risks [71]. After the 4-week text intervention, participants reported lower condom-unprotected sex events and more consistency with condom use at three months [71]. Another small pilot study reported successful engagement of rural women with opioid use disorder [82]. Researchers in that study noted that of those who used the sexual and reproductive health services, over a quarter did so for contraceptive care and 47% for pregnancy counseling or testing. However, only a fraction of those referred to in-person care attended an in-person visit, limiting the effectiveness of the intervention [82].

Telehealth has varied effectiveness for contraceptive uptake and long-term use. For those attending telehealth visits for contraceptive needs, there are positive long-term trends. One study measured long-term follow up for Depo Provera in young people between the ages of 13 and 21 years [28]. They found that those receiving the text message intervention were 3.65 more likely to be using Depo Provera 20 months after the end of the intervention. Similarly, another study provided subcutaneous Depo-Provera self-injection counseling via telehealth and 21% of participants opted to use that form of birth control [56]. One study noted that almost 70% of those who had a contraceptive telemedicine visit were still using the method chosen six months later, and 44% would choose a telemedicine visit for their next contraceptive appointment [79]. Additionally, in that same study, 76% of people referred for an in-person visit were seen in the clinic within 30 days for contraceptive follow-up [79]. For men seeking vasectomy, uptake was also not different between men who underwent telehealth consults for the procedure versus those who completed the consultation in person, highlighting the effectiveness of contraceptive counseling via telehealth [37]. However, when studying contraceptive uptake postpartum, the telehealth trends were not as significant. Some studies found minimal changes in contraceptive uptake in postpartum patients [19,21]. One study noted that patients in telehealth and in-person visits selected contraception at similar rates, although those in the telehealth group were less likely to choose long-acting reversible contraceptive or permanent sterilization, which would require in-person visits [21].

*Sexually transmitted infection prevention and treatment.* Seventy-five studies under KQ1 evaluated STI-related services for telehealth. Telehealth was found to be an effective mode of care in the delivery of STI prevention and treatment to numerous specific populations. One RCT evaluated telephone-delivered motivational interviewing to reduce risky sexual behavior in racial–ethnic minority women living with HIV [55]. They found that telephone-delivered motivational interviewing focused on individualized care significantly reduced sexual risk behavior, such as reducing substance use in sexual contexts and condomless sex. One RCT [86] and one mixed-methods study [30] both reported that telehealth services offering free specimen self-collection kits and telephone or video consultation effectively increased STI testing uptake and treatment among populations at high risk for contracting HIV. In one RCT that provided home STI test kits with video consultation for transgender youth, the level of PrEP use was very low, and sexual risk behavior did not change significantly over the follow-up period [75]. They reported that the participants were worried about having to be seen on camera by a person they did not know and felt hesitant to talk about sensitive topics [75]. In addition to telephone and video consultations, a few studies also showed that SMS/text-based intervention with a two-way communications component or with a telephone call component was effective in enhancing PrEP adherence among young men who have sex with men [42,58]. Their findings also demonstrated that the intervention was associated with a reduction in missed doses measuring by pill count [42]. Mobile health applications were also reported as effective in lowering the proportion of condom-unprotected sex in adolescent and young adult women with depressive symptoms and high-risk sexual behavior [71].

*Sexual wellness.* Five studies in this review explicitly reported sexual wellness outcomes in the context of effectiveness of telehealth interventions. These studies targeted special populations with different population-specific outcomes, which does not allow comparison across studies.

In a secondary analysis of an RCT for testing the efficacy of HIV tele-counseling in 424 men who have sex with men (MSM) couples, one study reported the impact of the intervention on the couples’ formation and adherence to safer sexual agreements [76]. Couples in the intervention (tele-counseling) arm had significantly greater odds of reporting a safer sexual agreement (3 months OR 1.87, *p*-value = 0.005, and 6 months OR 1.84, *p*-value = 0.007), lower odds of reporting discordant sexual agreements at 6 months (OR 0.62, *p*-value = 0.048), and a significantly lower odds of reporting breaking their sexual agreement (3 months OR 0.51, *p*-value = 0.035, and 6 months OR 0.23, *p*-value < 0.001). Another secondary analysis study of an RCT reported effects of a mobile app and a website based online discussion forum for reducing HIV stigma among young Black MSM over 6 months [23]. Participants who used the discussion forum reported an overall significant decrease in perceived HIV stigma (*p* < 0.05). Only participants with at least a high school education had a significant reduction in internalized homophobia over time (*p* = 0.006). Participants whose discussions focused on experiencing sexuality-related stigma reported increases in internalized homophobia (*p* ≤ 0.01) and sexual prejudice (*p* ≤ 0.05) over time. One UK-based study evaluated a virtual youth center for sexual minority young men to discuss HIV, sexuality, relationships, and sex [22]. In this study, 11 participants were guided through discussions to introduce new knowledge and build emotional resilience. All participants either maintained or improved their level of HIV knowledge and felt more confident about their sexuality (11% increase). One RCT in Turkey evaluated the effect of telephone counseling on the sexual lives of individuals with a bowel stoma following ileostomy or colostomy surgery [81]. Participants in the intervention arm reported significantly greater sexual satisfaction, reduced concern about sexual performance, and lower chances of lack of sexual desire 12 weeks after surgery (*p* < 0.01). One RCT evaluated a multi-component mHealth intervention focused on black gay youth with other vulnerabilities (e.g., polydrug use, alcoholism, anxiety and depression, experience of racial discrimination or sexual minority stigma) and reported no significant improvement in sexual risk behavior, i.e., self-reported number of condomless anal sex acts with serodiscordant and unknown sero-status partners in the past 3 months [121].

### 3.4. Key Question 2: Patient Experiences, Preferences, and Choice

*Contraceptive care and family planning.* Twenty-four studies under KQ2 addressed CC/FP. Overall, patients had positive experiences with contraceptive/family planning telehealth visits. Those who are already comfortable with telemedicine and direct-to-consumer advertisements are more comfortable using telehealth services [54]. Similarly, those who were less likely to value long-term relationships with providers were more likely to prefer telehealth [32]. A cross-sectional study at a tertiary care center in the USA reported that 54% of telemedicine patients reported “high-quality” contraceptive counseling compared to 64% of office visit patients (*p* = 0.29) [70]. Telehealth patients also reported ease of communication, less scheduling difficulty, overall convenience, efficiency with shorter visit times, and privacy as reasons for preferring telehealth visits [31,60,62,70]. Multiple studies note that patients were satisfied with the care they received and would consider using telehealth in the future [20,51,78], although one study noted that post-pandemic women found telehealth less acceptable than in-person care [62]. Those who preferred in-person visits over telehealth cite privacy, communication concerns, and perceived limits related to patient-centered care as rationale for preferences [51,57,70]. During the pandemic, one small RCT (*n* = 87) evaluating tele-counseling for contraceptive care in pregnant women in their third trimester reported patient preference favoring telehealth over in-person care for family planning counseling and education [147]

*Sexually transmitted infection prevention and treatment.* Fifteen studies under KQ2 addressed STIs. The majority of patients both in the United States and international countries report having positive experiences with telehealth in STI services. A small mixed-methods evaluation study in the United States reported that partnered gay, bisexual, or other men who have sex with men who participated in telehealth delivered via video chat for STI testing reported a very high level of service acceptability and quality [80]. Another small mixed-methods study focused on patients’ parents’ experience with a mobile health application to enhance HPV vaccination decision-making [25]. Parents rated the app as helpful and useful and that the parents would recommend the app to others [25]. Patients who received pre-exposure prophylaxis (PrEP) care via telehealth reported that the service was very helpful, confidential, fast, convenient, and easy to use, and they would recommend the method to others [58,67]. Similar to the study in the UK, patients were very satisfied with similar PrEP care via telehealth and reported that telehealth increased access to STI services at a lower cost [46,66]. Moreover, patients who received telehealth for STI services also reported that telehealth provides greater anonymity than usual care [49]. For people living with HIV, one large survey reported that during the peak pandemic period, a vast majority of patients preferred telemedicine over in-person care [155]. The main benefits of telemedicine (compared to in person) reported were savings of time and money, convenience, and ability to complete appointments as scheduled [155]. Just over half of PLHIV said they would feel more comfortable discussing sensitive topics (e.g., substance use, relationship issues) in person than over telephone (60%, *n* = 164) or video (55%, *n* = 151). Despite limited experience with video telemedicine, half of all participants desired a mix of telephone and video visits as part of their future HIV care [155].

*Sexual wellness.* Nine studies under KQ2 addressed sexual wellness. Two studies in this review focused on sexual wellness care services for adolescent populations through confidential, bi-directional, text message-based services. One study which focused on adolescents’ service preferences reported that they prefer interactive texting services that connect them to a person, although automated messages might be useful to bring new topics to their attention [85]. The other adolescent-focused study evaluated utilization and preferences for a confidential sexual health text-based helpline to expand Planned Parenthood of Western Pennsylvania’s sexual health programming [65]. This service was highly rated by users and was viewed as acceptable and appropriate. Common topics for information seeking among users included topics such as relationships, sexual acts, contraception, body image, STIs, pregnancy, values, Planned Parenthood services, sexual identity, and PrEP. One study reported that in the US, erectile dysfunction and contraception were among the leading reasons for utilization of direct-to-consumer telemedicine services [52]. Notably, in comparison to primary care physician visits, direct-to-consumer telemedicine services were used more frequently in urban areas and less frequently by low-income households [52]. For men’s sexual health, a large survey study in Texas (USA) reported that older patients were less likely to prefer telemedicine (odds ratio [OR], 0.55; 95% confidence interval [CI], 0.36–0.80; *p* < 0.001), less likely to agree to a video visit because of privacy concerns (OR, 0.51; 95% CI, 0.35–0.75; *p* < 0.001), and less likely to recommend a telemedicine visit compared with their younger counterparts (OR, 0.37; 95% CI, 0.27–0.51; *p* < 0.001); these preferences were regardless of distance from the andrology clinic, which was up to 57.5 miles [146]. One small RCT focused on post-menopausal women evaluated a mindfulness-based intervention via videoconferencing [153]. This RCT reported greater patient satisfaction and significant reduction in sexual distress scores but no significant improvement in sexual function scores [153]. For people living with HIV, one large survey conducted during the peak pandemic period reported that a majority of the patients expressed comfort with discussing sensitive topics such as relationship issues with care providers via telehealth [155].

### 3.5. Key Question 3: Provider Experiences and Preferences

*Contraceptive care and family planning.* Six studies assessed providers’ experiences with contraceptive/family planning telehealth consultations. One cross-sectional survey study reported that the majority of family planning providers believed telehealth visits were effective and should continue post-pandemic [77]. Conversely, another survey noted that the majority of healthcare providers were less satisfied with telehealth visits but would be willing to continue them in the future [62]. Another survey reported that among the clinicians interviewed for their study, all believed that telehealth was a good option, particularly for time-critical services and for patients living with disabilities, particularly in rural areas [31]. However, another survey study noted that approximately one quarter of physicians in their study expressed concerns for confidentiality for their adolescent patients [74].

*Sexually transmitted infection prevention and treatment.* Six studies discussed the providers’ experience with telehealth services for STI prevention or treatment; however, the feedback provided was generally positive. Due to the COVID-19 pandemic, a reduction in walk-in hours and in-person visits was reported [74]. Health providers rated telehealth platforms as excellent or good in delivering STI services in response to the rapid change from the pandemic [46]. They expressed that telehealth allowed them to assess patients safely and confidentially during the COVID-19 outbreak [46]. Some physicians noted that telehealth improved access for people with disabilities and those living in remote locations to services [31]. A small survey study in seven community-based organizations in two Southern states in the US reported that more than half of health providers working in community-based HIV/AIDS service organizations in the US were very interested in using mHealth apps to communicate and share general health tips with their patients in the future [84].

*Sexual wellness.* In this review, no studies related to sexual wellness evaluated provider perspectives or experience of telehealth services.

### 3.6. Key Question 4: Barriers and Facilitators for TeleSRH

*Contraceptive care and family planning.* Forty-four studies under KQ4 addressed CC/FP. During the pandemic, access to contraceptive/family planning telehealth visits drastically increased [20,33,74]. Patients without insurance were more likely to use telehealth services [57]. Barriers to telehealth for patients included not having a device [88], technology issues during the visit or concerns for miscommunication [51,70], or language discordance between the patient and provider [31]. In terms of miscommunication, an inability to read the providers’ body language or facial expressions emerged as a concern [31], while issues communicating with providers in general was cited as the reason to choose an in-person visit over telehealth [70]. Only one study found telehealth visit completion rates by race [87].

Multiple studies noted that telehealth visits facilitated care for underserved patient groups. For parenting teenagers and their families, telehealth filled a gap in reproductive and sexual health services [72]. Telehealth also connected rural-living women with opioid use disorders to care [82]. Participants in multiple studies noted that telehealth was particularly helpful when they lived at a distance from the clinic and had difficulty accessing care [32,51,82]. Additionally, other research teams found that patients chose telehealth visits to avoid a physical exam, to reduce wait times, and for privacy concerns [51,60].

Clinic-based barriers to providing telehealth stemmed from a lack of existing policies and procedures, reduced staff availability, and gaps in technology infrastructure [24,32]. There was an overall increase in the number of telehealth visits during the pandemic in states with insurance reimbursement, although it did not increase the overall number of contraceptive encounters [38]. Providers found that three quarters of contraceptive telehealth visits were covered by insurance, although this was slightly less than coverage for non-contraception visits (*p* < 0.001) [60]; however, interestingly, Medicare reimbursement was seen as a facilitator [24].

*Sexually transmitted infection prevention and treatment.* Forty-three studies under KQ2 addressed STIs. Although telehealth has been described as a silver lining in the midst of the COVID-19 outbreak, there were some significant challenges that limited the use of the telehealth method in STI services. Patients noted that technical difficulties and digital inequalities were barriers to delivering STI services via telephone [66]. Some patients, such as elderly people or people with economic disadvantages, may not have access to the internet or a phone/smartphone. Patients who were assessed for STI prescription via SMS text message were less likely to have an agreement with safe prescribing information than those assessed via telephone, and they were not likely to use the service in the future [61]. Patients were willing to use the telehealth platforms that they got used to, such as telephone, mobile applications, video calls, or SMS, for future service [31,84].

Patients’ and healthcare providers’ experiences and preferences with related technology were one of the facilitators of telehealth. According to clinicians and patients’ interviews, they felt that language barriers were a challenge faced by utilizing telephone consultation, particularly among new patients, leading to communication issues [31,66].

The current review noted some facilitators of telehealth methods for STI prevention and management services. Privacy and confidentiality were described as facilitators of using telehealth for STI services. A survey study in seven community-based organizations in two Southern states in the US reported that healthcare staff who were confident that safeguards are in place to keep electronically shared information from being seen by other people were very interested in using telehealth in future STI services [84].

*Sexual wellness.* Only a single study in this review evaluated any barriers for telehealth services for sexual wellness. This study reported that during the initial phase of the COVID-19 pandemic, compared to in-person care, adoption of telehealth services was lower among African Americans and multi-racial patients in Arkansas, Kansas, Missouri, and Oklahoma [48]. This study found no differences between adoption of telehealth or in-person care among Hispanic patients and reported an increase in adoption of telehealth services among White patients, as compared to in-person care during the initial phase of the COVID-19 pandemic. This study did not empirically evaluate any reasons to explain why race was a barrier to adoption of telehealth services during the initial phase of the pandemic [48].

### 3.7. Key Question 5: Patient Engagement Strategies

*Contraceptive care and family planning.* Eighteen studies under KQ5 addressed CC/FP. Engaging patients in contraceptive/family planning telehealth visits may depend on the patient’s comfort with technology. Those with higher levels of comfort with technology or prior experience with telehealth were more likely to choose a contraceptive/family planning telehealth visit [32,54]. Even with that comfort level, it is important to offer other accessible options to limit additional burden. One study effectively used telehealth visits in conjunction with mail order pharmacies and curbside contraceptive services, limiting the amount of time patients would spend on accessing care [33]. Another study provided regular text message reminders with case manager follow-up, resulting in a significant increase in contraceptive use at 20 months post-intervention [28]. Text message or smartphone alerts were used in other studies and showed patients were willing to engage with technology and were simultaneously satisfied with the intervention [69,71].

*Sexually transmitted infection prevention and treatment.* Twenty-four studies under KQ5 addressed sexual wellness. Multiple strategies were used to engage patients in telehealth for STI prevention and management services. Based on some studies found in this review, a possible strategy to enhance engagement of telePrEP includes weekly text/SMS messages with the option to set up reminders and a platform that supports two-way communication, such as telephone or video calls [30,42,58]. Video or telephone consultations were commonly utilized for STI testing with the services of free home test-kit delivery [30,36,67,75]. However, some patients prefer receiving text/SMS only for a negative result or phone calls to consult about the treatment if they receive a positive result [49]. To enhance STI/HIV-preventive attitudes, knowledge, and skills and reduce sexual risk behavior, the intervention commonly employs tailored telephone/video counseling that focuses on individualized care [29,59]. Additionally, standardized phone calls and mobile applications that provide information about vaccination with reminders and chat functions could be considered to be a good method for enhancing patient engagement in the HPV vaccination program [25,26].

*Sexual wellness.* Two studies in this review evaluated patient engagement strategies for telehealth [44,64]. One study evaluated videoconferencing as a strategy to deliver mindfulness-based intervention for sexual wellness among breast and gynecologic cancer survivors, the efficacy of which had been established before in in-person settings [44]. This study reported that adapting such an intervention for delivery via videoconferencing was feasible, perceived as appropriate, and acceptable among patients during the COVID-19 pandemic. In an adult population in Italy, one study evaluated patient engagement with a telephone helpline service between 2010 and 2017, which included sexual wellness counseling [64]. This study reported a decrease in requests over time for information about specific sexual dysfunctions, and general sexual health. Despite the advent of social media, there was an increase in the number of service users over the years, particularly among men who called most frequently for erectile dysfunction, suggesting that a telephone-based helpline may still be an important resource for patent engagement and delivery of sexual wellness care [64].

## 4. Discussion

There is relatively robust evidence that prescription of contraception via telehealth is feasible and acceptable to patients and providers. While comparable effectiveness (telehealth vs. in-person care) was not well established in the literature, patients generally found telehealth comparable in terms of acceptability and in many cases preferred telehealth over in-person contraception care. Studies on sexual wellness were scant and merit more research. The few studies on sexual wellness included in this review mostly reported positive results; however, this could potentially be due to publication bias. For contraceptive care/family planning services, we found only one small RCT (*n* = 87, Turkey) which evaluated effectiveness of telehealth for family planning counseling and education for third trimester pregnant women [147]. This RCT reported patient preference favoring telehealth over in-person care for counseling, education, and family planning consultation services [147]. We found no other RCTs evaluating telehealth for contraceptive care/family planning, but we found three quasi-experimental studies [51,56,82] and several observational studies which indicated that providing contraceptive care via telehealth is a feasible option in regard to clinical and patient-reported outcomes. By reducing barriers to care (e.g., transportation to clinics, childcare, etc.), telehealth provides methods of contraception to populations who may otherwise not receive consistent or any contraceptive care [31,72,82]. Arias et al. noted that long-acting reversible contraceptive methods may be selected less often by telehealth patients compared to patients seen in person [21]. However, telehealth patients receive contraceptive counseling on the full range of options and can select a long-acting reversible contraceptive, though it requires an in-person follow-up visit for placement.

The finding with perhaps the strongest evidence suggests that telehealth may be an important tool in the prevention of HIV. Two RCT articles reported on telehealth services to increase PrEP uptake and provide prescriptions among groups at high risk for contracting HIV [58,75]. These studies showed that telehealth may be an effective method of providing PrEP and/or promoting regular use. Similar results were found in four quasi-experimental studies [34,42,50,67] and two observational studies [46,66]. Some RCTs studies suggest that telehealth interventions may be an acceptable method of educating young people and their families about HPV vaccination [26,41,73,83]. The quasi-experimental study by Becker [25] further supports this finding. There were no observational studies that support these findings. However, there is not sufficient evidence to suggest that telehealth interventions are as effective or more effective in promoting HPV vaccine uptake compared to in-person interventions.

Although we did not conduct formal grading of evidence, our review team internally discussed the overall strength of the body of evidence following the GRADE/CERQual principles [161]. Overall confidence in the evidence on teleSRH is low to moderate, limited by study design, inconsistency, and indirectness. Most studies were observational and single-site, with heterogeneous telehealth models and outcome measures, leading to reduced confidence due to potential bias and limited comparability. Confidence in findings related to equity and quality of care was very low, reflecting incomplete and inconsistent reporting. Despite these limitations, the direction of the effect in the body of evidence was generally favorable, i.e., telehealth appeared to expand access and maintain safety and effectiveness across diverse SRH domains. However, uncertainty about the magnitude and durability of these effects remains. Future research should prioritize well-designed, adequately powered studies using standardized outcomes and equity-focused measures to strengthen certainty and guide sustainable, patient-centered implementation of teleSRH.

### 4.1. Applicability of Findings

Title X-funded clinics serve populations that differ markedly from the general U.S. SRH-seeking population. Title X clients are disproportionately low-income, racially and ethnically diverse, uninsured or underinsured, and more likely to experience structural barriers to obtaining in-person SRH care. Many rely on public transportation, shift-based employment, or caregiving responsibilities that constrain their ability to attend in-clinic visits. Telehealth may therefore offer unique value in this context by reducing logistical burdens and expanding access to contraception, STI services, and pregnancy-related care. The set of studies included in this review examined teleSRH interventions in study populations whose demographic characteristics (Supplemental Table S1) approximately resemble Title X patient profiles, including high representation of Black and Hispanic individuals, LGBTQ+ populations, and patients receiving care in safety-net or urban clinics.

It is also important to note that the countries represented in our review differ in their broader levels of telehealth adoption and digital health system maturity. For example, the U.S. is considered a high-capacity telehealth environment, yet adoption varies markedly across states, health systems, and reimbursement structures. Other very-high-HDI countries included in this review (e.g., Canada, Australia, Western and Northern European countries) have more uniform national telehealth frameworks, often supported by centralized governance and universal health coverage. The teleSRH interventions in our included studies therefore reflect localized programmatic implementation rather than national readiness. These differences underscore that while the synthesized evidence is relevant to U.S. Title X clinics, transferability may depend on local digital infrastructure, reimbursement pathways, and patient access to technology.

Several factors may influence the generalizability of our findings. First, sexual and reproductive health services are often provided alongside primary care, which means our review might not have encompassed all telehealth services related to sexual and reproductive health. Second, our review focused exclusively on high-income countries, with most of the studies originating from the United States. However, due to differences in healthcare and insurance systems, findings from other high-income countries may have limited and conditional applicability in US-based title X clinic settings, especially in regard to patient and provider experiences and barriers and facilitators to telehealth implementation. Relatedly, our choice to limit inclusion to very-high-HDI countries may have excluded informative teleSRH models from high-HDI settings with emerging digital health infrastructure. Expanding the geographic scope to include high-HDI countries (HDI 0.7–0.79) in future reviews could provide a broader perspective on telehealth implementation across diverse resource levels while still retaining relevance to U.S. Title X settings. Third, most of the studies included in the review were conducted during the COVID-19 pandemic, a period marked by a rapid expansion of telehealth services and the adoption of various communication methods between patients and providers. This resulted in a steep learning curve for healthcare systems, and initial data may not fully reflect the long-term effectiveness of telehealth services after the pandemic. Additionally, during the pandemic, even if some services were available via telehealth, many others were still disrupted; our findings are limited to the services covered by telehealth. Fourth, fewer studies on sexual wellness were identified for inclusion in the review, highlighting the need for caution when considering telehealth as a means to improve sexual wellness due to the limited amount of data available. Lastly, while this review included all accessible studies, it is important to acknowledge that there may be additional unpublished research related to telehealth in this domain that was not available at the time of our review.

### 4.2. Implications for Practice and Policy

This review has several important implications for clinical practice and policy development. The COVID-19 pandemic significantly disrupted clinical practice, making the use of technology for basic care essential [12]. Our findings show that patients are generally more open to telehealth for sexual and reproductive health services than healthcare providers. Given that telehealth is now integrated into many healthcare systems, providers must become more proficient in using telehealth tools. Health training programs should incorporate telehealth-specific training to prepare providers effectively. Moreover, clinics and hospitals must continue enhancing measures to protect patient confidentiality. Several studies raised concerns about confidentiality during sexual and reproductive health telehealth sessions, which could be compromised by either patients or providers [4,5,6]. Health systems should offer more guidance on maintaining confidentiality during these visits, such as ensuring private environments for both patients and providers. Documentation of how confidentiality was ensured should be a standard practice. Although telehealth is often seen as a solution for reaching rural or underserved populations, current research predominantly reflects experiences of urban populations. To improve telehealth accessibility in rural areas and for vulnerable groups, additional financial support is needed. Policy changes at the national and state levels will be essential to support these adjustments [12]. The 2010 Affordable Care Act (ACA) expanded access to sexual and reproductive health services, and the COVID-19 pandemic further accelerated telehealth adoption. Future research and funding should focus on evaluating telehealth outcomes, particularly among vulnerable populations, and assess its cost-effectiveness for health systems.

Of note, we recognize that teleSRH care exists within broader sociocultural and political contexts, including gender, sexuality, stigma, and structural inequities, that are beyond the scope of this implementation-focused review but remain critical areas for future research. Although AI is not within the scope of this review, it merits discussion. The use of AI in healthcare is accelerating at a time when AI use is not regulated [162]. There is an increasing use of virtual assistants, chatbots, and AI translation in healthcare services [162]. A lot of how those services work and how they are used is unclear to the patients [162,163]. The increasing adoption of AI technologies increases the risk of dehumanizing healthcare, eroding the already strained patient–provider relationship [163]. These are tools that should help the provider get closer to the patient, not tools replace the provider or create distance between the provider and the community. This would not benefit vulnerable communities and would increase the existing inequities [163]. As the AI landscape continues to evolve, integration of AI into telehealth warrants more research in regard to safety, efficacy, effectiveness, and provider + patient preferences.

## 5. Conclusions

This scoping review synthesizes a rapidly growing body of evidence on the use of telehealth in the delivery of sexual and reproductive health (SRH) services, revealing both promise and persistent challenges. Overall, telehealth demonstrates effectiveness and acceptability across key SRH service categories including contraceptive care/family planning, STI prevention and treatment, and sexual wellness, particularly in expanding access for underserved populations, including rural residents, adolescents, and individuals with stigmatized health needs. Patients largely report positive experiences, citing convenience, confidentiality, and efficiency, though preferences for in-person care remain among those valuing relational continuity or requiring procedures. Provider perspectives were generally supportive but reflect concerns about confidentiality and communication limitations, especially for adolescents and patients with language barriers. Barriers to telehealth include digital inequities, technology literacy, and infrastructural gaps, whereas facilitators include insurance coverage, flexible service modalities (e.g., text messaging, video, mail-order delivery), and tailored engagement strategies. Evidence on patient engagement strategies, such as SMS reminders, app-based counseling, and hybrid care models, suggests potential to enhance uptake and adherence to SRH care, though further study is needed to evaluate impact across diverse populations. Notably, sexual wellness remains understudied, particularly from the provider perspective. Overall, our findings underscore the need for policy and practice innovations that address structural and technological barriers, promote equity in digital access, and support the development of evidence-based, patient-centered telehealth models for SRH care. Future research should explore long-term outcomes, cost-effectiveness, and integration of telehealth into hybrid care models to ensure comprehensive, equitable delivery of sexual and reproductive healthcare in the post-pandemic landscape.

## Figures and Tables

**Table 1:** Populations, Interventions, Comparators, and Outcomes (PICOTS) and corresponding inclusion and exclusion criteria.

	Include	Exclude
Population	All Patient Populations	No Exclusions Based on Patient Population
Interventions	Studies related to delivery of sexual and reproductive health services via telehealth * Sexual and reproductive health services or interventions Sexual wellness education, counseling, and treatment Family planning (e.g., preconception/pre-pregnancy counseling, pregnancy testing and options counseling, basic infertility counseling) Contraception (e.g., screening, counseling, provision, and follow-up care) STI counseling, testing, and treatment	Telehealth * that is exclusively clinician-to-clinician communication Interventions without bi-directional communication between the patient and the healthcare team (e.g., one way email or text messages) Peer-led interventions (i.e., no clinician involvement) Pregnancy care Interventions or services that do not explicitly include family planning, contraception, STI prevention, or sexual wellness related care
Comparators	Studies related to effectiveness: Direct comparison between telehealth and usual care or in-person care or traditional care models (i.e., care provided without telehealth) Telehealth + in-person care (i.e., augmentation) vs. in-person care alone	No comparator-based exclusions.
Outcomes	All patient-related and provider-related outcomes relevant to key questions and interventions mentioned above	Outcomes not relevant to the key questions. Cost analyses
Timing	No restrictions of the timing of interventions and outcomes. Manuscript publication dates 2017–2025	Publication date prior to 2017
Study design	All primary research study designs involving quantitative and mixed-methods research.	Reviews of any type (e.g., systematic, scoping, narrative), qualitative research, non-research publications
Clinical setting	All healthcare settings: Home, outpatient, primary care, school-based health centers, or family planning clinics Clinics within prisons/jails Contact can be simultaneous (synchronous) or communicating across time (asynchronous) Individuals providing care include a broad range of healthcare workers (physicians, nurses, pharmacists, counselors, etc.) No geographic restriction; can be urban, suburban, or rural	Studies of healthcare services delivered outside of healthcare settings (e.g., social services, churches, exclusively academic settings)
Country setting	Countries with 2022 United Nations HDI designation of very high (0.8 or above)	Countries with 2022 United Nations HDI designation of less than very high (below 0.8)
Publication types	Peer-reviewed research studies	Editorial, commentary, non-peer-reviewed publication, any type of review article (e.g., systematic review)

Note: STI, sexually transmitted infection; HDI, human development index. * Telehealth includes interventions involving bi-directional communication between providers and patients using information and communication technology.

## Data Availability

The original contributions presented in this study are included in the article/Supplementary Material. Further inquiries can be directed to the corresponding author.

## Supplementary Materials

The following supporting information can be downloaded at https://www.mdpi.com/article/10.3390/clinpract16010014/s1. Table S1: Descriptive characteristics of included studies; Table S2: Summary of Findings; Table S3: Risk of bias assessments

## Appendix A

Key Definitions

Two key constructs were identified as focus points for this review: (1) telehealth and (2) sexual and reproductive health services.

*Telehealth*. In this manuscript, the term “telehealth” is used to define services that may include the use of information and telecommunications technology in healthcare delivery for a specific patient involving a provider across distance or time, such as remote real-time clinical visits and remote monitoring. For this review, we will refer to “telehealth” when considering interventions that use technology to facilitate interactions at a distance between specific patients and providers.

Interactions could occur over time (asynchronous) and over distance. We will consider telephone conversations, e-mail, and short message service (SMS) texts to be telehealth if they allow interaction between patient and provider and could replace or supplement an in-person interaction. These interventions will not be included if they occur only in one direction or if they are not personalized (e.g., phone, email or text message notifications; generic messages sent to a group of patients).

*Sexual and reproductive health services*. In this manuscript, sexual and reproductive health (SRH) services considered for this review include contraceptive care, family planning, sexually transmitted infection (STI) prevention and treatment services, and sexual wellness. For this review, contraceptive care includes screening, counseling, provision of contraception, and follow-up care. Family planning services include pre-conception/pre-pregnancy counseling (including birth spacing), pregnancy testing and options counseling, and basic infertility counseling. STI prevention and treatment services include STI counseling, testing, treatment, and prophylaxis, including Human Papilloma Virus (HPV) vaccination counseling and education. Sexual wellness services include education, counseling and treatment related to sexual self-esteem, sexual satisfaction, sexual awareness, and body esteem. We will consider sexual and reproductive health services that can be delivered via telehealth by a broad range of healthcare workers (e.g., physicians, nurses, pharmacists, counselors).

## Appendix B

Search Strategy

**Ovid MEDLINE(R) and Epub Ahead of Print, In-Process, In-Data-Review & Other Non-Indexed Citations and Daily <25 January 2025>**

1. exp Reproductive Control Agents/
2. exp Contraception Behavior/or exp Contraception/
3. (contracept* or birth control).mp.
4. exp Family Planning Services/
5. family planning.mp.
6. exp Gynecology/or exp Women’s Health/
7. (gyn?ecolog* or (women* adj2 health)).mp.
8. exp reproductive health/or exp sexual health/
9. ((reproduct* adj health*) or (sexual* adj health*)).mp.
10. exp Sexually Transmitted Diseases/
11. (sexually transmitted disease* or sexually transmitted infection* or STD or STDs or STI or STIs).mp.
12. exp Fertility/
13. fertility.mp.
14. exp Abortion, Induced/
15. abortion*.mp.
16. exp pregnancy, unplanned/or exp pregnancy, unwanted/
17. ((unplanned or “not planned” or unwanted or “not want*”) adj3 pregnan*).mp.
18. ((sexual or reproductive) adj2 (wellness or wellbeing or well-being)).mp.
19. (preconception or pre-conception or prepregnancy or pre-pregnancy).mp.
20. exp Papillomavirus Vaccines/or (papillomavirus vaccin* or HPV vaccin* or gardasil or papillomavirus immunis* or papillomavirus immuniz* or HPV immunis* or HPV immuniz*).mp.
21. or/1–20
22. (telemedicine or telemedical or telehealth or telephone or phone or phones or smartphone* or cell* device* or mobile device* or text messag* or SMS or texting or virtual* or remote* monitor* or ehealth or e-health or mhealth or m-health or mobile health or digital health).ti.
23. exp *Telemedicine/or exp *Mobile Applications/or exp *Cell Phone/
24. 22 or 23
25. 21 and 24
26. limit 25 to (English language and yr = “2017-Current”)

**Embase**

1. exp contraceptive agent/or exp contraceptive behavior/or exp contraception/or birth control/
2. (contracept* or birth control).mp.
3. exp family planning/
4. family planning.mp.
5. exp gynecology/or exp women’s health/
6. (gyn?ecolog* or (women* adj2 health)).mp.
7. exp reproductive health/or exp sexual health/
8. ((reproduct* adj health*) or (sexual* adj health*)).mp.
9. exp sexually transmitted disease/
10. (sexually transmitted disease* or sexually transmitted infection* or STD or STDs or STI or STIs).mp.
11. exp fertility/
12. fertility.mp.
13. exp induced abortion/or exp abortive agent/
14. abortion*.mp.
15. exp unplanned pregnancy/or exp unwanted pregnancy/
16. ((unplanned or “not planned” or unwanted or “not want*”) adj3 pregnan*).mp.
17. ((sexual or reproductive) adj2 (wellness or wellbeing or well-being)).mp.
18. (preconception or pre-conception or prepregnancy or pre-pregnancy).mp.
19. exp Human papilloma virus vaccine/or (papillomavirus vaccin* or HPV vaccin* or gardasil or papillomavirus immunis* or papillomavirus immuniz* or HPV immunis* or HPV immuniz*).mp.
20. or/1–19
21. (telemedicine or telemedical or telehealth or telephone or phone or phones or smartphone* or cell* device* or mobile device* or text messag* or SMS or texting or virtual* or remote* monitor* or ehealth or e-health or mhealth or m-health or mobile health or digital health).ti.
22. exp *telehealth/or exp *mobile application/or exp *mobile phone/
23. 21 or 22
24. 20 and 23
25. limit 24 to (english language and yr = “2017-Current”)
26. limit 25 to conference abstract status
27. 25 not 26

**CINAHL**

S1. (MH “Reproductive Control Agents+”)
S2. (MH “Contraception+”)
S3. (contracept* OR birth control)
S4. (MH “Family Planning+”)
S5. family planning
S6. (MH “Gynecology”) OR (MH “Gynecologic Nursing”) OR (MH “Gynecologic Care”) OR (MH “OB-GYN Nurse Practitioners”)
S7. (gyn#ecolog* OR (women* N2 health))
S8. (MH “Reproductive Health”) OR (MH “Sexual Health”)
S9. ((reproduct* N1 health*) OR (sexual* N1 health*))
S10. (MH “Sexually Transmitted Diseases+”)
S11. (sexually transmitted disease* OR sexually transmitted infection* OR STD OR STDs OR STI OR STIs)
S12. (MH “Fertility+”)
S13. fertility
S14. (MH “Abortion, Induced+”)
S15. abortion*
S16. (MH “Pregnancy, Unplanned”) OR (MH “Pregnancy, Unwanted”)
S17. ((unplanned OR “not planned” OR unwanted OR “not want*”) N3 pregnan*)
S18. ((sexual OR reproductive) N2 (wellness OR wellbeing OR well-being))
S19. (preconception OR pre-conception OR prepregnancy OR pre-pregnancy)
S20. (MH “Papillomavirus Vaccine”)
S21. (papillomavirus vaccin* OR HPV vaccin* OR gardasil or papillomavirus immunis* OR papillomavirus immuniz* OR HPV immunis* OR HPV immuniz*)
S22. S1 OR S2 OR S3 OR S4 OR S5 OR S6 OR S7 OR S8 OR S9 OR S10 OR S11 OR S12 OR S13 OR S14 OR S15 OR S16 OR S17 OR S18 OR S19 OR S20 OR S21
S23. (MM “Telehealth+”)
S24. (MM “Mobile Applications”)
S25. (MM “Cellular Phone+”)
S26. TI (telemedicine OR telemedical OR telehealth OR telephone* OR phone OR phones OR smartphone* OR cell* device* OR mobile device* OR text messag* OR SMS OR texting OR virtual* OR remote* monitor* OR ehealth OR e-health OR mhealth OR m-health OR mobile health OR digital health)
S27. S23 OR S24 OR S25 OR 26
S28. S22 AND S27
